# Spatial proteomics reveal that the protein phosphatase PTP1B interacts with and may modify tyrosine phosphorylation of the rhomboid protease RHBDL4

**DOI:** 10.1074/jbc.RA118.007074

**Published:** 2019-06-07

**Authors:** Kyojiro N. Ikeda, Matthew Freeman

**Affiliations:** Dunn School of Pathology, University of Oxford, Oxford OX1 3RE, United Kingdom

**Keywords:** phosphoproteomics, rhomboid protease, intramembrane proteolysis, proteostasis, protein–protein interaction, polyubiquitin chain, tyrosine-protein phosphatase (tyrosine phosphatase), endoplasmic-reticulum-associated protein degradation (ERAD), BioID, Lys^63^-linked ubiquitin, PTP1B PTP1N, rhomboid-like protein 4 (RHBDL4), VCP p97

## Abstract

Rhomboid-like proteins are evolutionarily conserved, ubiquitous polytopic membrane proteins, including the canonical rhomboid intramembrane serine proteases and also others that have lost protease activity during evolution. We still have much to learn about their cellular roles, and evidence suggests that some may have more than one function. For example, RHBDL4 (rhomboid-like protein 4) is an endoplasmic reticulum (ER)–resident protease that forms a ternary complex with ubiquitinated substrates and p97/VCP (valosin-containing protein), a major driver of ER-associated degradation (ERAD). RHBDL4 is required for ERAD of some substrates, such as the pre–T-cell receptor α chain (pTα) and has also been shown to cleave amyloid precursor protein to trigger its secretion. In another case, RHBDL4 enables the release of full-length transforming growth factor α in exosomes. Using the proximity proteomic method BioID, here we screened for proteins that interact with or are in close proximity to RHBDL4. Bioinformatics analyses revealed that BioID hits of RHBDL4 overlap with factors related to protein stress at the ER, including proteins that interact with p97/VCP. PTP1B (protein-tyrosine phosphatase nonreceptor type 1, also called PTPN1) was also identified as a potential proximity factor and interactor of RHBDL4. Analysis of RHBDL4 peptides highlighted the presence of tyrosine phosphorylation at the cytoplasmic RHBDL4 C terminus. Site-directed mutagenesis targeting these tyrosine residues revealed that their phosphorylation modifies binding of RHBDL4 to p97/VCP and Lys^63^-linked ubiquitinated proteins. Our work lays a critical foundation for future mechanistic studies of the roles of RHBDL4 in ERAD and other important cellular pathways.

## Introduction

Rhomboids are evolutionarily conserved intramembrane serine proteases (1). They belong to the superfamily of rhomboid-like polytopic membrane proteins that includes active proteases and the enzymatically inactive pseudoproteases. Although the function is only known for a subset of rhomboid-like proteins, there is a clear theme that they control the fate of membrane proteins in the secretory pathway, in the mitochondria, and at the plasma membrane. To date, this role manifests in a variety of biological contexts including parasite infection, inflammatory responses, bacterial quorum sensing, growth factor secretion, mitochondria, and proteostasis (2–12). As membrane proteins, the rhomboid-like proteins have modular structures, with cytoplasmic, transmembrane, and luminal/extracellular domains, and there are emerging suggestions that some members of this superfamily might have more than one function.

The endoplasmic reticulum (ER)^3^ resident rhomboid protease RHBDL4, which is conserved in metazoans and plants (8), exemplifies this apparent multifunctionality. Recent reports indicate roles for RHBDL4 in canonical rhomboid-like release of a single pass TM protein, amyloid precursor protein (13); in secretion of transforming growth factor α (TGFα) as full length in exosomes (14); and, best understood, in controlling proteostasis by ER-associated degradation (ERAD) (8). RHBDL4 binds to p97/VCP, a AAA+-type ATPase that powers ERAD, as well as binding to ubiquitinated substrates (8, 15). The assembly of this complex drives the cleavage and targeting to ERAD of the model substrate pTα, the α-chain of the progenitor form of the T-cell receptor (8, 16). Similarly, RHBDL4 determines ERAD targeting of a major adhesion molecule of the peripheral nervous system, myelin protein zero (8). More recently, RHBDL4 has been reported to trigger the release of the epithelial growth factor (EGF) receptor ligand pro-TGFα in a process that is distinct from canonical rhomboid transmembrane domain cleavage of growth factors (14). In this case, full-length pro-TGFα is released associated with exosomes from cells, by a mechanism that is currently unknown, although its traffic and secretion dynamics is likely controlled by RHBDL4 catalytic residues. Finally, in another context, RHBDL4 behaves as a sheddase-like rhomboid protease, cleaving and thereby releasing the extracellular domain of the amyloid precursor protein (APP), and its close homologues, amyloid precursor like protein 1 (APLP1) and 2 (APLP2), in a cholesterol-regulated manner (13, 17). This event can be influenced by the primary sequence of the substrate transmembrane region (18), is distinct from the one performed by α-secretase, and might avoid subsequent toxic processing by β- and γ-secretases. All these diverse cases depend on the conserved catalytic residues Ser^144^ and His^195^ that comprise the rhomboid catalytic dyad. However, despite this dependence on protease activity, the molecular mechanisms underlying these functions remain unknown and appear to be very different from each other, perhaps representing distinct molecular roles of RHBDL4.

Post-translational modifications diversify the functions of proteins. Regulatory modifications such as phosphorylation may therefore be responsible for determining the appropriate action of multifunctional proteins. For example, phosphorylation triggered by G protein–coupled receptor stimulation with agonists such as phorbol 13-myristate 12-acetate was found to alter activity of members of the pseudoprotease iRhom2. This facilitates the trafficking of tumor necrosis factor α–converting enzyme (TACE/ADAM17) in a serine/threonine phosphorylation–regulated manner, with the help of the phosphosite-binding 14-3-3 protein (5, 19–21). Similarly, phorbol 13-myristate 12-acetate treatment can synergize with RHBDL4 activity to increase the secretion of TGFα (14). Until now there have been no reports of whether RHBDL4 is regulated by any post-translational modification that could affect its molecular functions.

A powerful approach to uncovering the functional mechanisms of uncharacterized proteins is to identify their binding partners. BioID is a spatial proteomic approach in which a promiscuous R118G mutant of the *Escherichia coli* biotin ligase BirA (BirA*) is used to label proteins within the radius of ∼10 nm from the bait (22–25). Because of a biotin-streptavidin–based isolation strategy combined with stringent washes with 2.0% SDS, BioID provides a restricted list of candidate neighbors. Unlike more classical co-immunoprecipitation approaches, BioID can identify very weak and transient interactions that may nevertheless be functionally important including, for example, enzymes such as kinases or E3 ligases and their substrates (26, 27). To begin to understand how RHBDL4 can play its distinct roles, we conducted a comparative spatial proteomic study using BioID. We performed a BioID screen for RHBDL4, using the pseudoprotease iRhom2 as a comparative negative control to help identify RHBDL4-specific partners. Notable RHBDL4 hits included the nonreceptor type tyrosine phosphatase PTP1B, known also as PTPN1. As an initial approach to validating their functional significance, we used MS to identify a cluster of phosphorylated tyrosine residues in the RHBDL4 cytoplasmic tail and uncovered their ability to modulate binding to Lys^63^-linked polyubiquitin and p97/VCP. This work begins to address the molecular protein networks surrounding RHBDL4 and therefore to provide a foundation for future mechanistic understanding of how this conserved rhomboid protease performs its apparently diverse biological roles.

## Results

### Establishment of BioID of RHBDL4 and iRhom2

**Figure 1.**
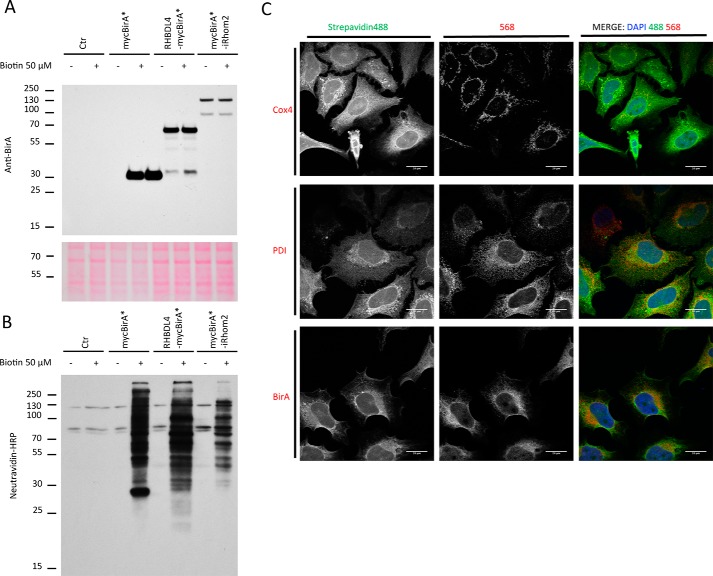
**Characterization of RHBDL4 and iRhom2 BioID cell lines.** *A*, HEK293 control cell lines, otherwise stably expressing mycBirA*, RHBDL4mycBirA*, or mycBirA*iRhom2 were treated or not with 50 μm biotin for 24 h. The cells were lysed and probed with antibody against BirA. HEK293 cells free of mycBirA* were used as controls. As a loading control, Ponceau S red staining of the same membrane is shown. *B*, lysates of HEK293 cell lines stably expressing mycBirA*, RHBDL4mycBirA*, or mycBirA*iRhom2 were treated with biotin 50 μm for 24 h, lysed, and probed with neutravidin-HRP. *C*, HeLa cells stably expressing RHBDL4mycBirA* were labeled using anti-BirA, PDI, and COX4 antibodies and compared by confocal light microscopy to streptavidin-488 labeling. Size marker is 20 μm. *Ctr*, control; *DAPI*, 4′,6′-diamino-2-phenylindole.

We performed a BioID screen with the protease RHBDL4 and, as a negative control to assess the specificity of RHBDL4 hits, with iRhom2, a nonprotease rhomboid-like protein located predominantly in the ER. WT human RHBDL4 was tagged at the cytoplasmic C terminus with a flexible arm of seven serines followed by BirA* (RHBDL4mycBirA*). Likewise, human iRhom2 was tagged at its cytoplasmic N terminus with mycBirA* followed by seven serines (mycBirA*iRhom2). We generated HeLa cells stably expressing mycBirA*, RHBDL4mycBirA*, and mycBirA*iRhom2. Additionally, we made HEK293 cells stably expressing RHBDL4mycBirA*. The expected sizes of the tagged proteins were measured by Western blotting as mycBirA* c30 kDa, RHBDL4mycBirA* c70 kDa, and mycBirA*iRhom2 c130 kDa (Fig. 1*A*). These molecular masses are consistent with those of BirA (35 kDa), RHBDL4 (36 kDa), or iRhom2 (93 kDa). Treatment with 50 μm biotin for 18 h had no impact on these bands (Fig. 1*A*), but when neutravidin-HRP was used to detect cellular biotinylation, the same lysates showed intense labeling in biotin treated-cells that were expressing mycBirA*, RHBDL4mycBirA*, and mycBirA*iRhom2 (Fig. 1*B*). As expected, endogenous mitochondrial carboxylases that contain biotin as covalently bound co-factor are visible in all cells at 130 kDa and between 100 and 70 kDa. In immunofluorescent micrographs, RHBDL4mycBirA* and mycBirA*iRhom2 were primarily localized in the ER, which is consistent with the localization of RHBDL4 and iRhom2 (8, 28) (Fig. 1*C* and Fig. S1). mycBirA*iRhom2 but not RHBDL4mycBirA* also showed occasional nuclear localization when localized with anti-BirA* antibody (Fig. S1). Upon treatment with biotin, RHBDL4mycBirA*-dependent biotinylation was detected with fluorescent streptavidin-Alexa 488 conjugate, and comparing it with the mitochondrially localized carboxylases that were labeled by anti-cytochrome *c* oxidase 4 (COX4) antibody (Fig. 1*C*, *top row*). Interestingly, biotinylation was also detected toward the periphery of the cell, not overlapping with the ER marker, phosphodiester isomerase (PDI), or RHBDL4mycBirA*, which was localized with anti-BirA antibody. We concluded that the localization of RHBDL4mycBirA* and mycBirA*iRhom2 is consistent with the literature, and the biotinylation levels induced upon treatment with biotin were compatible with proteomic use.

### Bioinformatic analysis of BioID show enrichment in protein-stress related factors

**Figure 2.**
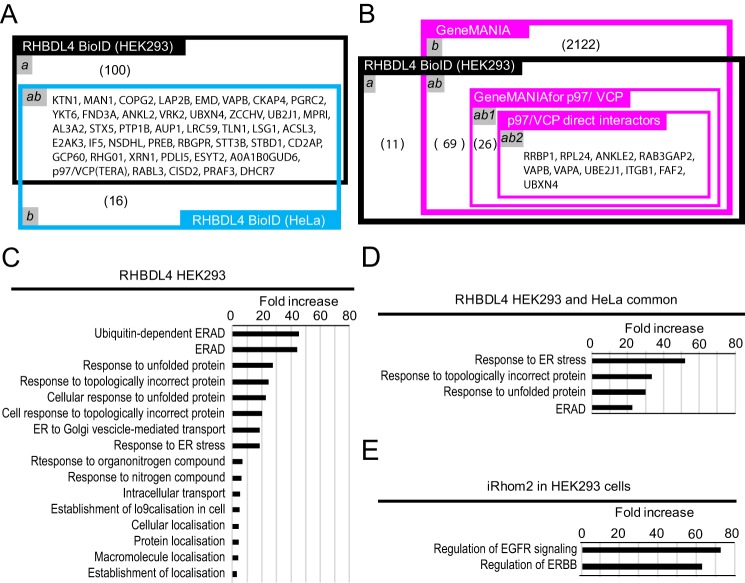
**Analysis of BioID-identified candidates.** *A* and *B*, values in *brackets* represent the number of proteins that correspond to the Venn domain. *A*, Venn diagram showing the overlap between candidates identified by the BioID of RHBDL4 in HEK293 cells (*black*) with HeLa cells (*blue*). *a*, number of proteins that are exclusive to the BioID of RHBDL4 in HEK293 cells. *ab*, proteins that are common between the BioID of RHBDL4 in HEK293 cells and HeLa cells. *b*, proteins that are only in the BioID of RHBDL4 in HeLa cells. *B*, Venn diagram showing the overlap of identifications from the BioID of RHBDL4 in HEK293 cells (*black*) with known interactors of p97/VCP using GeneMANIA and Cytoscape 3 (*magenta*). *a*, number of proteins unknown to GeneMANIA. *ab1*, number of proteins in the BioID of RHBDL4 in HEK293 cells that are found to be linked to VCP/p97 in GeneMania. *b*, proteins present in GeneMANIA that do not overlap with the BioID. *ab*, generic proteins that are present in GeneMANIA and overlap with RHBDL4 BioID in HEK293 cells. *ab1*, proteins in the BioID of RHBDL4 in HEK293 cells that are known indirect interactors of p97/VCP to GeneMANIA. *ab2*, proteins in the BioID of RHBDL4 in HEK293 cells that are physical direct interacting with p97/VCP, according to GeneMANIA. *C*, fold enrichment of Gene Ontology function terms with respect to expected frequency, after PANTHER search in BioID of RHBDL4 in HEK293 cells *D*, fold enrichment of GO terms among common candidates between RHBDL4 BioID in HEK293 cells and HeLa cells. *E*, fold enrichment of GO terms in the BioID of iRhom2 in HeLa cells.

Biotinylated proteins in extracts from cells expressing mycBirA* alone, RHBDL4mycBirA*, and mycBirA*iRhom2 were captured by affinity purification and analyzed by MS. Results from two independent replicates in HEK293 cells and three replicates of HeLa cells were compared. We excluded any protein with less than three weighted spectral counts in all lists. We also ignored any protein that was found in the mycBirA* alone condition, because we considered them non-specific hits of the mutant biotin ligase (R118G). In HEK293 cells, peptides mapped reproducibly to more than 146 proteins for RHBDL4 that were absent in the BioID of BirA* alone (Fig. 2*A*, *domains a* and *ab*, and Table S1). BioID was repeated in HeLa cells, where ∼60 reproducible candidates were identified for RHBDL4 (Fig. 2*A*, *domains ab* and *b*, and Table S1). By intersecting the BioID of RHBDL4 from HEK293 and HeLa, 43 proteins were identified as common and high priority hits (Fig. 2*A*, *domain ab*, and Table S1). Similarly, ∼28 candidates specific for iRhom2 were identified after exclusion of proteins that are in common with the BioID of mycBirA* or RHBDL4 (Table S1). Approximately 80 proteins were common hits between RHBDL4 and iRhom2 in the BioIDs that were performed in HEK293 (Table S1).

GeneMANIA is an algorithm that compares available proteomic, transcriptomic, genetic, shared domain, and predicted interactions to any given list of proteins to draw possible interaction networks (29–31). We used GeneMANIA application in the data analysis software Cytoscape 3.6.1 that enables further analysis of networks. We constructed a gene network using the 146 identifications of the RHBDL4 HEK293 BioID and compared this to previously published proteomic and genetic studies (32–38) (Fig. S2 and Table S2). Of these 146 identifications, 104 were available on GeneMANIA; 35 of 146 proteins could be linked to p97/VCP using all the search criteria of the GeneMANIA networks (Fig. 2*B*, *domain ab1*), whereas 10 were related to p97/VCP via evidence of direct interactions in proteomic or candidate-based studies (Fig. 2*B*, *domain ab2*). In addition, among the hits of BioID of RHBDL4 in HEK293 and HeLa cells, there are known interactors of VCP/p97. For instance, the ERAD factor UBXN4 interacts with p97/VCP to reduce misfolded proteins in *Caenorhabditis elegans* and *Homo sapiens* (39). The B-cell chronic lymphocytic leukemia-related E3 ubiquitin-ligase TRIM13 (TRI13) is an interactor of p97/VCP (40); similarly the deubiquitinase involved in postmitotic Golgi reassembly VCPIP1 interacts with VCP/p97 and the Golgi SNARE co-factor STX5 (41–43).

In a complementary approach to predicting gene function, we used the Gene Ontology (GO) PANTHER classification system (44–46). GO includes the molecular functions, cellular components, and biological processes associated with genes by the GO Consortium. PANTHER searches through GO terms associated with a given protein and provides an estimation for the enrichment of specific GO terms within a list of proteins, with respect to a randomized control list. From the list of BioID hits of RHBDL4 in HEK293 cells, the algorithm identified ∼144 proteins with mostly ER-related functions as enriched by at least 2-fold with respect to expected frequency (Fig. 2*C* and Table S3). Proteins associated with membrane localization and insertion, response to unfolded proteins or topologically incorrect proteins, and ERAD were clearly enriched over 10-fold compared with expected frequency (Fig. 2*C*). Similarly, this enrichment was maintained, with at least a 30-fold increase in frequency, when the 43 reproducible common hits of the BioIDs of RHBDL4 in HeLa and HEK293 cells were searched (Fig. 2*D*). By comparison, the 28 reproducible candidates in the BioID of iRhom2 scored highest for functions related to multivescicular bodies and EGF signaling with at least 60-fold increase frequency and no predicted enrichment for ER functions (Fig. 2*E*). Importantly, both BioID of RHBDL4 and iRhom2 contained previously described interactors. For example, p97/VCP is found consistently in the BioID of RHBDL4, and TACE/ADAM17 is in the BioID of iRhom2 (Table S1).

### RHBLD4 is substrate of the nonreceptor type tyrosine phosphatase PTP1B, also called PTPN1

The PANTHER search on the BioID of RHBDL4 in HEK293 cells highlighted roles in topology-related stress functions. We analyzed the predicted topology of the BioID candidate list for RHBDL4 in HEK293 and HeLa cells and compared with the BioID of iRhom2 in HeLa cells. RHBDL4 BioID in HEK and HeLa cells contained 10–20% of proteins with C-terminal TM domain proteins). As expected, we found increased presence of TM domain proteins (∼60%) in all BioID of RHBDL4. Among the 146 proteins that are found only in the BioID of RHBDL4 in HEK cells, 16 contain a single TM domain at the N terminus and 21 at the C-terminal domain (25%). In the BioID of RHBDL4 in HeLa cells, proteins with terminal TM domain are 21 (34%). On the contrary, no protein with terminal TM domain was in the list of proteins exclusive to the BioID of iRhom2 in HEK cells (Fig. S3*A* and Table S1). Among the 43 common candidates identified in RHBDL4 BioID in both HEK293 and HeLa cells, there are 18 protein (40%) with TM at either N or C terminus. Among those, 7 are tail anchor (TA) proteins: LAP2B, VAPB, FND3A, VRK2, AL3A2, STX5, and PTP1B (Fig. S3*B* and Table S1).

**Figure 3.**
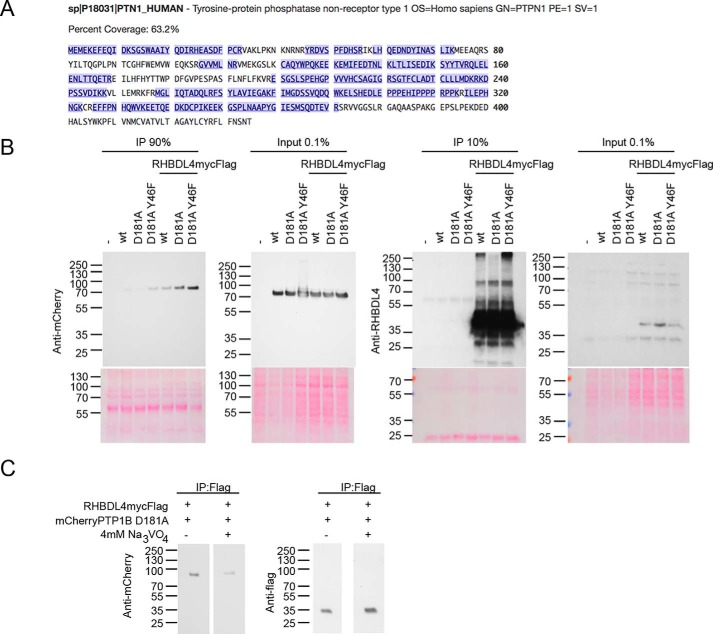
**Identification of PTP1B (also called PTPN1) in the BioID and as interactor of RHBDL4.** *A*, peptide overage of PTP1B in the BioID of RHBDL4. Peptides identified by MS are shown in *blue. B*, the substrate-trapping mutant D181A and the super-trapping mutant D181A/Y46F of mCherry-PTP1B were transiently expressed with or without RHBDL4mycFLAG. The cells were lysed in immunoprecipitation (*IP*)–compatible conditions and captured with anti-FLAG immunomagnetic beads. Co-immunoprecipitates and whole cell lysates (*Input*) were probed with anti-mCherry, or RHBDL4 antibody. *C*, HEK293 cells expressing TMEM115-mycFLAG or RHBDL4mycFLAG were lysed and bound with anti-FLAG beads ± 4 mm Na_3_VO_4_. The resulting co-immunoprecipitates were probed with anti-FLAG-HRP or anti-mCherry antibody.

The tyrosine phosphatase PTP1B (also called PTPN1) is an ER resident TA protein with roles in ER stress and recycling of receptors (47). PTP1B is common to the BioID of RHBDL4 in both HEK293 cells and HeLa cells and is a lower hit in the BioID of iRhom2 in HEK293 cells (Fig. 3*A*). PTP1B is a clinically relevant treatment target for cancer and diabetes with roles in insulin and EGF signaling and anxiety (48–51). In GeneMANIA, 21 genes in the BioID of RHBDL4 in HEK293 cells had predicted direct connections with PTP1B. In particular, ITGB1 are direct physical interactors of both PTP1B and p97/VCP (Table S2). Overall, PTP1B is a well-connected protein to VCP/p97 and other hits within the BioID of RHBDL4, and we hypothesized that it could be a significant member of the RHBDL4 protein network.

To test the possibility that RHBDL4 is a substrate of PTP1B and is therefore itself tyrosine-phosphorylated, we used substrate-trapping mutants of the phosphatase. The enzymatic mechanism of PTP1B is well-characterized and a substrate-trapping mutant (D181A) as well as a super-trapping mutant (D181A/Y46F) with even higher affinity to substrates have been previously described (52–55). Such mutants are needed to identify possible substrates because the interaction between a WT enzyme and its substrate is typically too transient. WT, D181A, and D181A/Y46F mutants of mCherry-PTP1B were expressed in the absence or presence of co-transfected RHBDL4, C-terminally tagged with myc and FLAG (RHBDL4mycFLAG). In co-immunoprecipitations using anti-FLAG beads, mCherry-PTP1B WT and mutants D181A and D181A/Y46F were detected by anti-mCherry antibody, weakly for WT PTP1B, more strongly for the D181A mutant, and most strongly for the super-trapping D181A/Y46F (Fig. 3*B*). This interaction was inhibited by the tyrosine phosphatase inhibitor, Na_3_VO_4_ at a concentration of 4 mm. When this competitive inhibitor was present in washes, we observed reduced binding of mCherry-PTP1B to RHBDL4mycFLAG (Fig. 3*C*). These results suggest that RHBDL4 could be a substrate of the tyrosine phosphatase PTP1B.

### Proteomic identification of phosphorylations on RHBDL4

**Figure 4.**
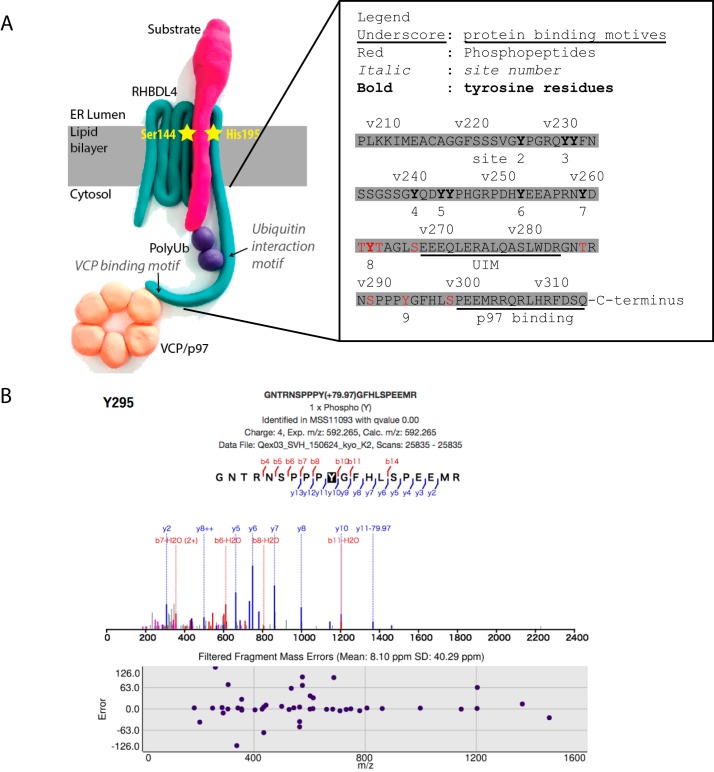
**Mapping of identified post translational modifications to the C terminus of RHBDL4.** *A*, plasticine model of RHBDL4 interactions with mapping of phosphopeptides to the primary sequence of the C terminus (209–315) of RHBDL4. In *red* are identified phosphorylations, and in *bold type* are the 11 tyrosine residues. Site 1 (Tyr^205^) is predicted to be in the transmembrane domain and was ignored. Site 2 (Tyr^227^), site 3 (Y233F/Y234F), site 4 (Y242F), site 5 (Y245F/Y246F), site 6 (Y254F), site 7 (Tyr^261^), site 8 (Tyr^264^), and site 9 (Tyr^295^) are represented in *bold type. B*, analysis of the fragmentation of the peptide GNTRNSPPPYGFHLSPEEMR encompassing Tyr^295^ and the filtered fragment mass error (*q* value < 0.01).

Our observation of RHBDL4 binding to PTP1B (also called PTPN1) substrate-trapping mutants led us to investigate directly whether RHBDL4 is itself tyrosine-phosphorylated. The analysis of the last 110 amino acid residues of the C terminus of RHBDL4, which is its major cytoplasmic domain, contains 10 tyrosine residues that are organized into eight sites, where two sites are tyrosine doublets (Fig. 4*A*). An additional tyrosine, Tyr^205^, is predicted to be part of the last transmembrane domain. The tyrosine residues of the C terminus of RHBDL4 are close to important interaction motifs capable of binding to ubiquitin and p97/VCP (8). Tyrosine frequency in the proteome is ∼3%, compared with ∼10% in this RHBDL4 cytoplasmic domain (206–315). Similar enrichment of tyrosine residues exists in the C terminus of RHBDL4 homologues of different vertebrates including *Mus musculus*, *Gallus gallus*, *Xenopus tropicalis*, and *Danio rerio* (Fig. S4). Consistent with the conservation of these tyrosine residues, MS analysis of the BioID data for RHBDL4 revealed phosphorylated peptides in the C terminus of RHBDL4. Specifically, residues Thr^263^/Tyr^264^/Thr^265^, Ser^269^, Thr^289^, Ser^291^, Tyr^295^, and Ser^300^ (Fig. 4, *A* and *B*, and Fig. S5*B*) were predicted to include phosphate groups. For example, in the peptide ^290^NSPPP**Y**GFHLSPEEMR^305^, Tyr^295^ was identified with gain of mass +79.97 compatible with a phosphorylation detected between fragments b10 and y11 (*q* ≤ 0.01) (Fig. 4*B*). In other cases, ^290^NSPPP**Y**GFHLSPEEMR^305^ was phosphorylated at Tyr^295^ in 21 of 123 peptides with *q* ≤ 0.01, whereas ^259^NYDT**Y**TAGLSEEEQLER^276^ was phosphorylated in residue Thr^263^, Tyr^264^, or Thr^265^ in 11 of 32 peptides with *q* ≤ 0.01.

### Tyrosine residues of the C terminus of RHBDL4 are important for interaction with p97/VCP and Lys^63^-linked ubiquitin

**Figure 5.**
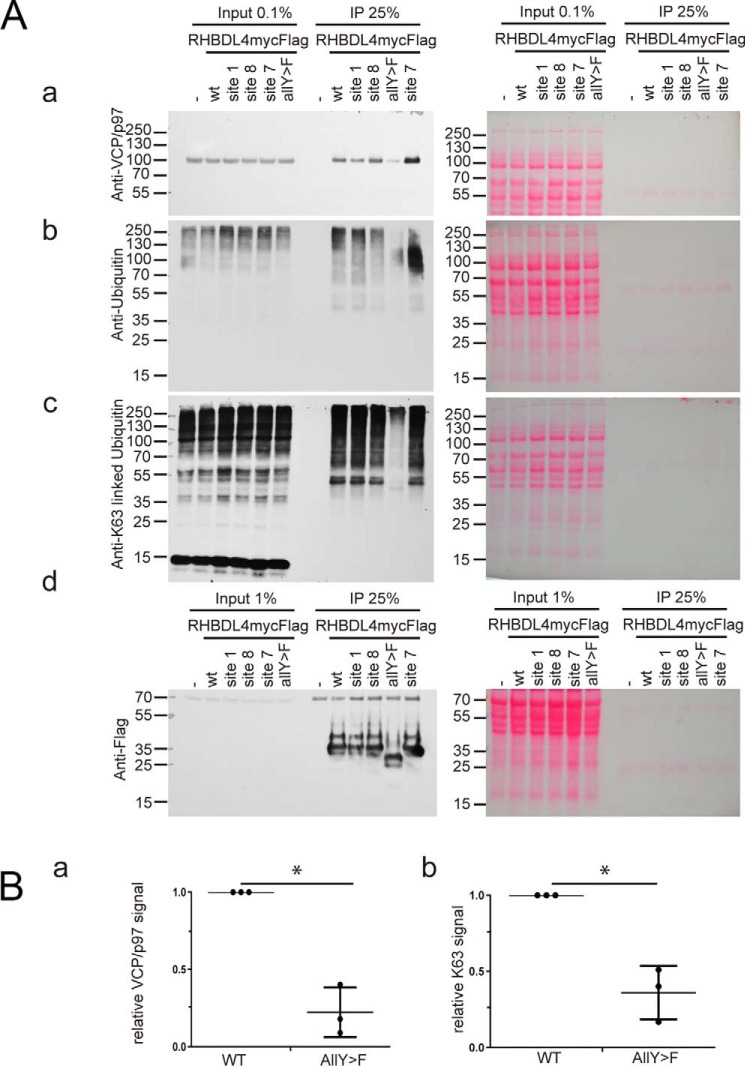
**Co-immunoprecipitation (*IP*) of Lys^63^-linked ubiquitin and p97/VCP with mutants of RHBDL4.** HEK293 cells expressing myc-FLAG–tagged mutants of RHBDL4 for site 1 (Y206F), site 7 (Y261F), site 8 (Y264F), and all-sites mutant were lysed and incubated with anti-FLAG beads. Co-immunoprecipitates and whole cell lysates (*Input*) were probed with anti-p97/VCP (*panel a*), anti-ubiquitin (*panel b*), anti-Lys^63^ linkage–specific antibodies (*panel c*), and anti-FLAG-HRP for RHBDL4mycFLAG (*panel d*). *B*, relative quantification of the immunoreactivity of co-immunoprecipitates (*panel a*) *versus* anti-p97/VCP (*, *p* = 0.014, *n* = 3) (*panel b*) or anti-Lys^63^-linked ubiquitin (*, *p* = 0.024, *n* = 3) of WT RHBDL4mycFLAG and AllY→F, normalized against the input. The *error bars* represent standard deviation from the means.

We tested whether these tyrosines influence the binding of p97/VCP and ubiquitin. The mutants of individual sites or a mutant in which all tyrosines are replaced by phenylalanines (AllY→F) did not affect cellular distribution, and we detected no major effect on cellular morphology when observed by confocal light microscopy; all mutants showed similar expression levels and localization (data not shown). The variants of RHBDL4mycFLAG were affinity-captured using anti-FLAG beads and compared with WT RHBDL4. Conversely, the mutant AllY→F showed reduced binding to p97/VCP when co-immunoprecipitates were probed with anti-p97/VCP antibody (Fig. 5, *A*, *panel a*, and *B*, *panel a*). This mutant runs faster on a gel than the WT or mutants for individual sites (Fig. 5*A*, *panel d*). Interestingly, the site 7 mutant (Y261F) appears to show slightly increased binding to p97/VCP. In addition to the altered interactions with p97/VCP, the AllY→F mutant showed strongly reduced binding to endogenous ubiquitin (Fig. 5*A*, *panel b*) and to Lys^63^-linked polyubiquitin (Fig. 5, *A*, *panel c*, and *B*, *panel b*). The site 7 (Y261F) mutant might have altered binding of endogenous ubiquitin but was undistinguishable concerning the binding of Lys^63^-linked ubiquitin (Fig. 5*A*, *panels b–d*). Overall, these results show that C-terminal tyrosine residues of RHBDL4 are important for binding RHBDL4 to ubiquitin, Lys^63^-linked ubiquitin and p97/VCP.

## Discussion

In this work, we present the first spatial proteomics performed on a member of the rhomboid-like protein superfamily. Using spatial proteomics approaches like BioID, it is possible to detect both strong interactions that can be found by conventional methods but also weaker and more transient proximal proteins, which may nevertheless be functionally important. Using BioID, we probed the protein neighborhood of RHBDL4, comparing it with iRhom2 as a control for the specificity of any detected interactions. GeneMANIA network analysis indicated that the RHBDL4 BioID hits partially overlapped with candidate interaction lists for p97/VCP and PTP1B (also called PTPN1) from previously performed proteomic interaction screens. These earlier studies were done using affinity purification–based strategies, and the presence of common candidates helps to validate our BioID screen on RHBDL4. TMHMM analyses showed that BioID of RHBDL4 contains TA proteins, and intriguingly, PTP1B is a TA protein too. It will be interesting to investigate in the future the potential interplay between PTP1B, other identified TA proteins, and RHBDL4. For instance, Vamp-associated protein (VAP) homologues are present in the BioID of RHBDL4. VAPA and VAPB are TA proteins and important adaptor proteins of the ER with roles at ER contact sides and has been recently associated with PTP1B activity in regulating receptor recycling (56–59).

PANTHER Gene Ontology analysis indicates enrichment in the BioID of RHBDL4 for terms related to ERAD, protein stress at the ER, and protein localization. Conversely, terms related to EGF signaling are enriched in the BioID of iRhom2. This is consistent with the current view of RHBDL4 as an ERAD determinant (8), with roles as a secretase enzyme (14, 60), whereas iRhom2 regulates EGF signaling by regulating TACE/ADAM17, a major sheddase of EGF receptor ligands (28). Similarly, the enrichments for protein localization GO terms in the BioID of RHBDL4 are consistent with the roles in the control of traffic dynamics that have been suggested (14). From this point of view, it is intriguing that BioID of RHBDL4 identified PTP1B, a tyrosine phosphatase related to ER stress and recycling of signaling receptors (47).

The identification of the tyrosine phosphatase PTP1B led us to discover that the C terminus of RHBDL4 is indeed tyrosine-phosphorylated. Moreover, we have shown that the binding of p97/VCP and ubiquitin is affected by mutations targeting these tyrosines, providing the first indication that RHBDL4 may be regulated by tyrosine kinases. This will be an important area for future exploration. We also found that RHBDL4 binds to Lys^63^-linked polyubiquitin and that this interaction is also reduced when all tyrosine residues in the C terminus are mutated to phenylalanines. Unlike Lys^48^-linked ubiquitin, which is more often related to proteasome functions (61), Lys^63^-linked ubiquitin has been related to autophagy, protein traffic and signaling (61, 62). This result provides another platform for future investigation of RHBDL4 function. An E3 ubiquitin ligase, TRIM13, was also identified in the RHBDL4 BioID hits. TRIM13 is an ER-localized E3 ubiquitin ligase that can facilitate the degradation of CD3δ (40). Additionally, a role for TRIM13 in ER-stress induced autophagy has been suggested (63, 64). On the other hand, overexpression of this E3 ligase correlates to increased Lys^29^-linked polyubiquitin (65), indicating that it is premature to build functional hypotheses on this possible interaction, which will need further investigation.

In the past, BioID had been successful in identifying novel candidates that are hard to detect by more conventional strategies such as affinity purification. The BioID of RHBDL4 further strengthens the value of this technology, although our list of proteins might be skewed by the expression of additional copies of RHBDL4. We report here many interesting candidates for functional partners of RHBDL4, and our work provides the foundations for targeted mechanistic investigations of how these putative interactors may act with RHBDL4 in its emerging roles in cellular quality control, protein traffic, and signaling. Furthermore, the identification of phosphosites at the C terminus of RHBDL4 and tyrosine residues that are important for p97/VCP and ubiquitin binding suggests new regulatory mechanisms. For instance, RHBDL4 is required for G protein–coupled receptor–stimulated TGFα release in exosomes (14). Finally, this work validates the use of BioID as a tool to investigate functionally relevant proteins that interact with the rhomboid-like proteins more generally, as well as other factors in the secretory pathway.

## Experimental procedures

### Cloning and generation of BioID cell lines

cDNA of RHBDL4 and iRhom2 were PCR-amplified from fibroblast cDNA library “Basinger preparation” kindly provided by Chris Norbury. 7 serine flexible arm (7S) was introduced between mycBirA* and the bait protein by overlap PCR. The cDNAs of RHBDL4–7S-mycBirA* and mycBirA*-7S-iRhom2 so generated were introduced by gateway cloning (Thermo Fisher Scientific) into FU-tetO-Gateway (Addgene, 43914) via DONR221, a plasmid kindly provided by Dragana Ahel. HeLa and HEK293 cells were transfected using Lipofectamine and selected with 400 μg of Zeocin initially and then kept at 200 μg/ml.

### Western blots

NuPAGE precast gradient 4–12% polyacrylamide (Thermo Fisher Scientific) was used for anti-BirA blot. All other blots are either 10% or 12.5% polyacrylamide:bis (37.5:1) gels. The membranes were incubated in 5% milk in PBS with Tween or Nonidet P-40 at 0.2%. Anti-RHBDL4 (Sigma, HPA013972) was used at 1:200. Anti-K63 linkage–specific (Abcam, EPR8590-448), anti-p97 (Thermo Scientific Pierce), anti-mCherry (GeneTex, GTX128508), and anti-myc clone 4A6 (Merck Millipore, 05-724) antibodies were all used at 1:1000. Anti–FLAG-HRP (Sigma–Aldrich A8592) was used at 1:2000 whenever indicated. Neutravidin blots were done after blocking 3% BSA in PBS, with 0.2% Tween 20; neutravidin–HRP conjugate (Thermo Fisher Scientific) was used at 1:4000 in 3% BSA in PBS. 0.1% Ponceau S stain in 1% acetic acid was used to stain nitrocellulose membranes.

### Immunofluorescence

The cells on cover glasses were rinsed in PBS three times prior to fixation in paraformaldehyde 4% in PBS for 20 min. The fixative was substituted twice with 200 mm PIPES with 100 mm glycine and incubated for 40 min. Fixed cells were extracted with 0.2% Triton X-100 in PBS for 20 min. The cover glasses were postfixed in methanol at −20 °C for 45 min and then rehydrated in 3% BSA in PBS at 4 °C overnight. The next day, the cover glasses were incubated with primary antibodies diluted in 3% BSA with 0.2% Triton X-100 in PBS for 2 h. Dilution of the primary antibodies is 1:100 for PDI (Cell Signaling, 3501), 1:500 for COX4 (Cell Signaling, E311), 1:800 for anti-BirA (Abcam, ab14002), and 1:800 for streptavidin-488 (Thermo Fisher Scientific). The cover glasses were washed in 3% BSA three times. Anti-mouse, -rabbit, or -chicken goat antibody 568 was used at 1:2000 in 3% BSA for 1 h as secondary labeling. Cover glasses were mounted on slides on 4′,6′-diamino-2-phenylindole mount (ProLong^TM^ Diamond antifade mountant; Thermo Fisher Scientific) and then sealed with nail polish and air-dried. Confocal light micrographs were taken at an Olympus FV1000 at 63× or 100× magnification.

### Image analysis

Quantifications of intensities of signals from Western blotting or images of Ponceau red stains were done using Nikon D3200 camera and ImageQuant (GE Healthcare). All *p* values are calculated by using Student's *t* tests on values from three independent biological replicates. The *error bars* are the standard deviation from the mean. Models were made with plasticine (Play-Doh, Hasbro), then photographed with MotoG5 cell phone camera, and edited with Photoshop (Adobe). All immunomicrographs were processed with Fiji/ImageJ and postprocessed in Photoshop (Adobe).

### BioID and MS

Stably expressing BioID cell lines were treated with biotin 50 μm for 24 h prior to lysis in ice-cold radioimmune precipitation assay buffer with cOmplete protease inhibitor (Roche). The sample was then added with 2× final modified Laemmli's buffer (120 mm Tris-HCl, pH 6.8, 4% SDS, 0.8% 2-mercaptoethanol, and 20 μm 1,4-DTT, 4% glycerol, 0.05% bromphenol) supplemented with biotin 50 μm and boiled for 10–15 min at 95 °C. For proteomic submission, cell culture was scaled to two 15-cm plates per condition. Biotinylated proteins were isolated using original Roux-buffer system for BioID (22); Only modification has been the use of 1–2.5 kU of Benzonase (Sigma–Aldrich), prior to addition of immunomagnetic MyOne streptavidin C1 beads (Lifesciences). Moreover, biotinylated proteins were eluted from beads in modified Laemmli's buffer just mentioned, without glycerol and bromphenol but supplemented with biotin 50 μm at 95 °C for 9.5 min. 10% of eluates were kept for later neutravidin-blot analysis, prior to submission for MS analysis. Remaining 90% was processed by filter-aided sample preparation (66). The sample was washed 5–13 times in 8 m urea in 100 mm triethylammonium bicarbonate (TEAB), pH 8.5, before reduction by 10 mm tris(2-carboxyethyl)phosphine in 8 m urea in 100 mm TEAB, pH 8.5. Tris(2-carboxyethyl)phosphine was removed, and the sample was alkylated with 10 mm chloroacetamide in 8 m urea for 30 min. Alkylated material was washed with 8 m urea in 50 mm TEAB, pH 8.5, and resuspended into 1 m urea in 50 mm TEAB, pH 8.5. The sample was tryptically digested at a 1:5 enzyme:substrate ratio at 37 °C overnight and then blocked with formic acid 1%. Peptides were eluted in 0.1% TCA and 50% acetonitrile in 0.1% TCA. These peptides were speed vacuumed to dryness, prepurified from particulate with 100% acetonitril in 0.1% TCA equilibrated handmade C18 custom columns, and eluted in 50% acetonitrile in 0.1% TCA. Finally, the sample was resuspended in 0.1% formic acid in 2% acetonitrile and sonicated in ultrasonic bath. For injection purposes, the peptides were resuspended in 5% formic acid and 5% DMSO and then trapped on a C18 PepMap100 pre-column (300-μm inner diameter × 5 mm, 100 Å; Thermo Fisher Scientific) with 0.1% formic acid, at 26 bar and separated on an Ultimate 3000 UHPLC system (Thermo Fisher Scientific) coupled to a QExactive mass spectrometer (Thermo Fisher Scientific). The peptides were separated on an in-house packed analytical column (360 μm × 75-μm inner diameter packed with ReproSil-Pur 120 C18-AQ, 1.9 μm, 120 Å; Dr. Maisch GmbH) and then electrosprayed into an QExactive mass spectrometer (Thermo Fisher Scientific) through an EASY-Spray nano-electrospray ion source (Thermo Fisher Scientific) using a linear gradient (length, 60 min, 15% to 35% solvent B (0.1% formic acid in acetonitrile); flow rate, 200 nl/min). The raw data were acquired on the mass spectrometer in a data-dependent mode. Full-scan MS spectra were acquired in the Orbitrap (scan range, 350–2000 *m*/*z*; resolution, 70,000; AGC target, 3e6; maximum injection time, 50 ms). After the MS scans, the 10 most intense peaks were selected for HCD fragmentation at 30% of normalized collision energy. HCD spectra were also acquired in the Orbitrap (resolution, 17,500; AGC target 5e4; maximum injection time, 120 ms) with first fixed mass at 180 *m*/*z*.

### Bioinformatic analyses of BioID

The results were analyzed via Central Proteomics Facilities Pipeline (67) and triple searched in MASCOT (68) Server (Matrix Sciences), X!Tandem (69) kscore (GPM), and Open Mass Spectrometry Search Algorithm (70). These triple-searched peptides were matched against UPR_HomoSapiens. Searches were conducted with one maximum missed trypsin cleavage, fixed carbamidomethyl with variable cysteine, biotinylated lysine and N terminus, or methionine oxidation as post-translational modifications. Precursor tolerance was at 20 ppm, and fragment tolerance was at 0.1 Da, with 1+, 2+, and 3+ default charge states. Weighted spectral counts were quantified with SINQ (71) with *q* value of ≤0.01. Identifications with less than three spectral counts were manually discarded and then ranked according to the sum of spectral counts of biological replicates. Common candidates from different experiments were searched via a custom-made Python coded application. From so-obtained lists of reproducible candidates, gene networks were generated using GeneMANIA and Cytoscape 3.6.1 (29–31); we compared the candidate list of BioIDs against the previously published proteomes as in Refs. 30 and 32–37. Direct interactions were selected using Cytoscape → MANIA tools → physical interaction → select first neighbors and then table panel → export table. GeneMANIA-predicted-missing-link candidates were removed manually. Circular disposition of results was chosen to represent Fig. S2. Potential biological functions of identified candidates were searched using the Gene Ontology PANTHER Classification System (44–46) (http://pantherdb.org/),^4^ and GO terms with more than 2-fold increase in frequencies are reported in the results. Prediction of the topology of the BioID hits were performed by batch retrieval of the primary sequences of protein in FASTA format via Uniprot (https://www.uniprot.org/batch/).^4^ Sequences so obtained were batch uploaded on TMHMM server 2.0 (www.cbs.dtu.dk/services/TMHMM/).^4^ We scored manually the presence of predicted TM domain with in 50AA from either N or C termini of proteins Finally, we used the definition of TA proteins from Borgese *et al.* (72) to score their presence.

### Site-directed mutagenesis and transfections

All point mutations were introduced by site-directed mutagenesis using CloneAmp HiFI PCR premix (Clontech). pCMV6 RHBDL4mycFLAG was purchased from Origene (RC210708). C termini of AllY→F mutant was designed with Geneart (Thermo Fisher Scientific) and used as long primers to PCR using WT RHDBL4 as template. The PCR product was cloned into SgfI/XhoI–excised RC210708 plasmid, by Gibson assembly (NEB). mCherry-PTP1B D181A was obtained from Addgene (40270). mCherry-PTP1B WT was generated by reverting the Ala^181^ codon back to aspartate, whereas super-trapping mutant D181A/Y46F was generated by site-directed mutagenesis of Tyr^46^ to Phe on the D181A mutant (Addgene, 40270).

### Co-immunoprecipitations

HEK293 cells were grown to 70% confluency on 15-cm dishes. The cells were transfected with 2 μg of DNA for all constructs using Lipofectamine Ltx at 1% with plus reagent at 0.5% (Thermo Fisher Scientific). After 8.5 h, fresh medium was provided. After 24 h, the cells were lysed in ice-cold 1% Triton in 50 mm Tris, pH 7.4, with 150 mm NaCl, cOmplete protease inhibitors, 2 mm sodium pervanadate, and 1 mm sodium fluoride. Sodium pervanadate was prepared by repeated heating and adjustment of alkalinity to pH 10 with NaOH. Lysates were centrifuged for 10 min at 1000 × *g* at 4 °C. Supernatants were collected and provided with 250 units of benzonase before addition to pre-equilibrated immunomagnetic anti-FLAG M2 beads (Sigma–Aldrich). Lysates were incubated with beads for 90 min and then washed in same lysis buffer four times for 5 min for mCherry-PTP1B variants. In washout experiments, Na_3_VO_4_ was added to the wash buffer at 4 mm final. Additional 100 mm NaCl was provided to the wash buffer to co-immunoprecipitates that were eluted in above mentioned modified Laemmli's buffer for later probing with anti-p97VCP or anti-ubiquitin antibodies.

## Author contributions

K. N. I. and M. F. conceptualization; K. N. I. and M. F. resources; K. N. I. data curation; K. N. I. software; K. N. I. formal analysis; K. N. I. and M. F. supervision; K. N. I. and M. F. funding acquisition; K. N. I. validation; K. N. I. investigation; K. N. I. visualization; K. N. I. methodology; K. N. I. and M. F. writing-original draft; K. N. I. and M. F. project administration; K. N. I. and M. F. writing-review and editing; K. N. I. all experimental work.

## Supplementary Material

Supporting Information

## Footnotes

4 Please note that the JBC is not responsible for the long-term archiving and maintenance of this site or any other third party hosted site.

## Footnotes

3 The abbreviations used are:

ER: endoplasmic reticulum
ERAD: ER-associated degradation
TGF: transforming growth factor
TM: transmembrane
EGF: epithelial growth factor
GO: Gene Ontology
VAP: Vamp-associated protein
PDI: phosphodiester isomerase
TEAB: triethylammonium bicarbonate.
