# Supplementary material for: Spatial proteomics reveal that the protein phosphatase PTP1B interacts with and may modify tyrosine phosphorylation of the rhomboid protease RHBDL4
Source: J Biol Chem. 2019 Jun 7;294(30):11486–97. doi: 10.1074/jbc.RA118.007074 (PMC6663880; doi:10.1074/jbc.RA118.007074)
Supplement: Supporting Information [file supp_294_30_11486__index.html]

Spatial proteomics reveal that the protein phosphatase PTP1B interacts with and may modify tyrosine phosphorylation of the rhomboid protease RHBDL4 — BioID of RHBDL4 finds phosphorylations and PTP1B interaction — Spatial proteomics reveal that the protein phosphatase PTP1B interacts with and may modify tyrosine phosphorylation of the rhomboid protease RHBDL4 — BioID of RHBDL4 finds phosphorylations and PTP1B interaction — Supporting Information 

# Spatial proteomics reveal that the protein phosphatase PTP1B interacts with and may modify tyrosine phosphorylation of the rhomboid protease RHBDL4

## Supporting Information

- Combined Supporting Information file
- Table S1 (to be published online) - BioID of RHBDL4 and iR2
- Table S2 (to be published online) - GeneMANIA Cytoscape analysis of the BioID
- Table S3 (to be published online) - Table S3 contains PANTHER Go term search of the BioID
