# Supplementary material for: Spatial proteomics reveal that the protein phosphatase PTP1B interacts with and may modify tyrosine phosphorylation of the rhomboid protease RHBDL4
Source: J Biol Chem. 2019 Jun 7;294(30):11486–97. doi: 10.1074/jbc.RA118.007074 (PMC6663880; doi:10.1074/jbc.RA118.007074)
Supplement: Supporting Information [file supp_RA118.007074_142469_2_supp_344665_psqsmt.pdf]

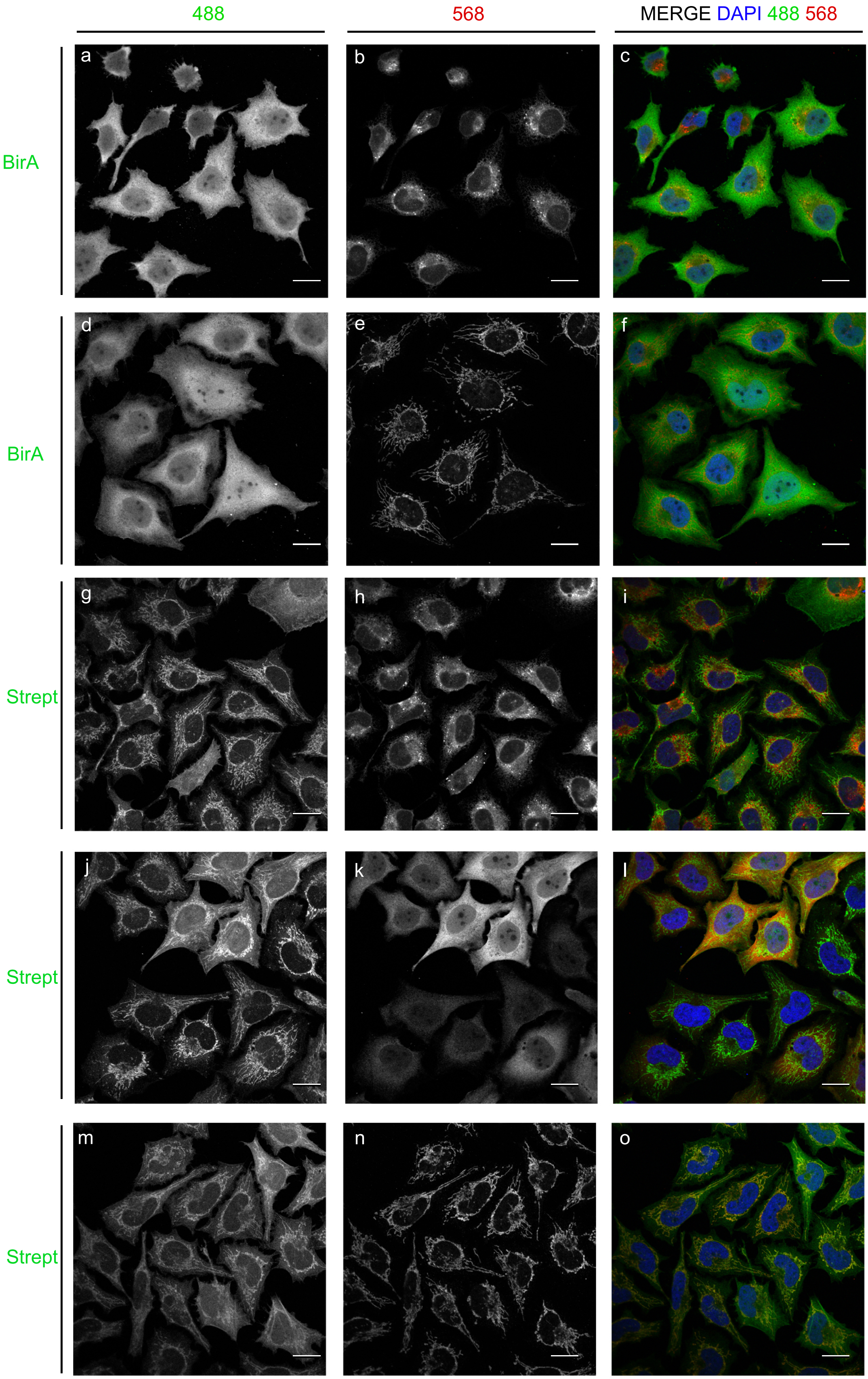

Supporting Information 1 HeLa cells stably expressing mycBirA*iRhom2 were prepared for immunofluorescence and stained with anti-BirA, Streptavidin-488, anti-PDI, anti-COX4, and DAPI prior to imaging with confocal microscopy. Size markers represent 20μm in anti-BirA immunofluorescent micrographs (a-f). For Streptavidin-488 biotin localisation micrographs (g-o), size bar is 32 μm.

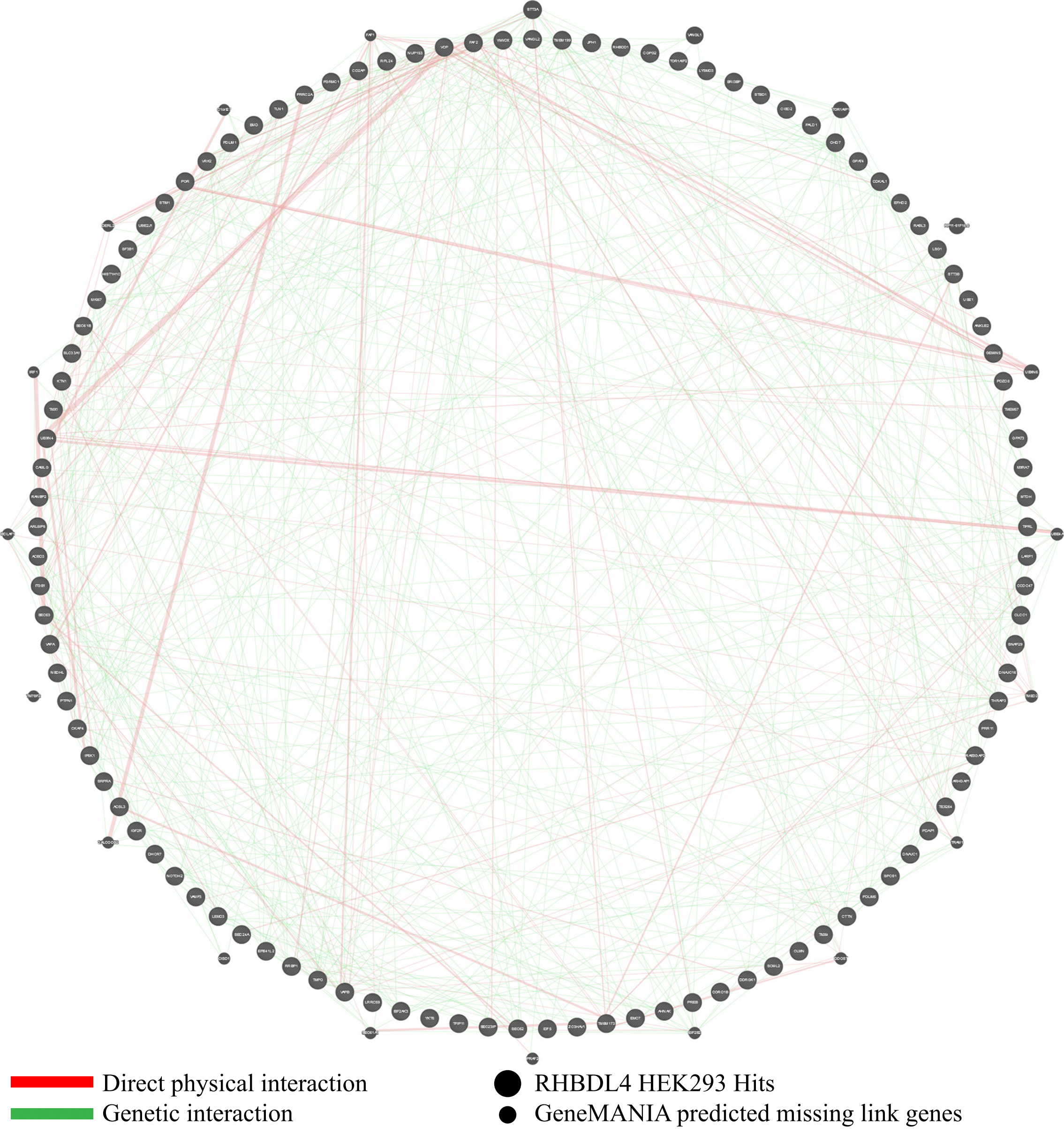

Supporting Information 2 GeneMANIA-specified network of genetic (green) and physical (red) interactions of the BioID of RHBDL4 in HEK293 cells. Large circles are actual BioID hits, while small circles are GeneMANIA predicted missing links among proteins.

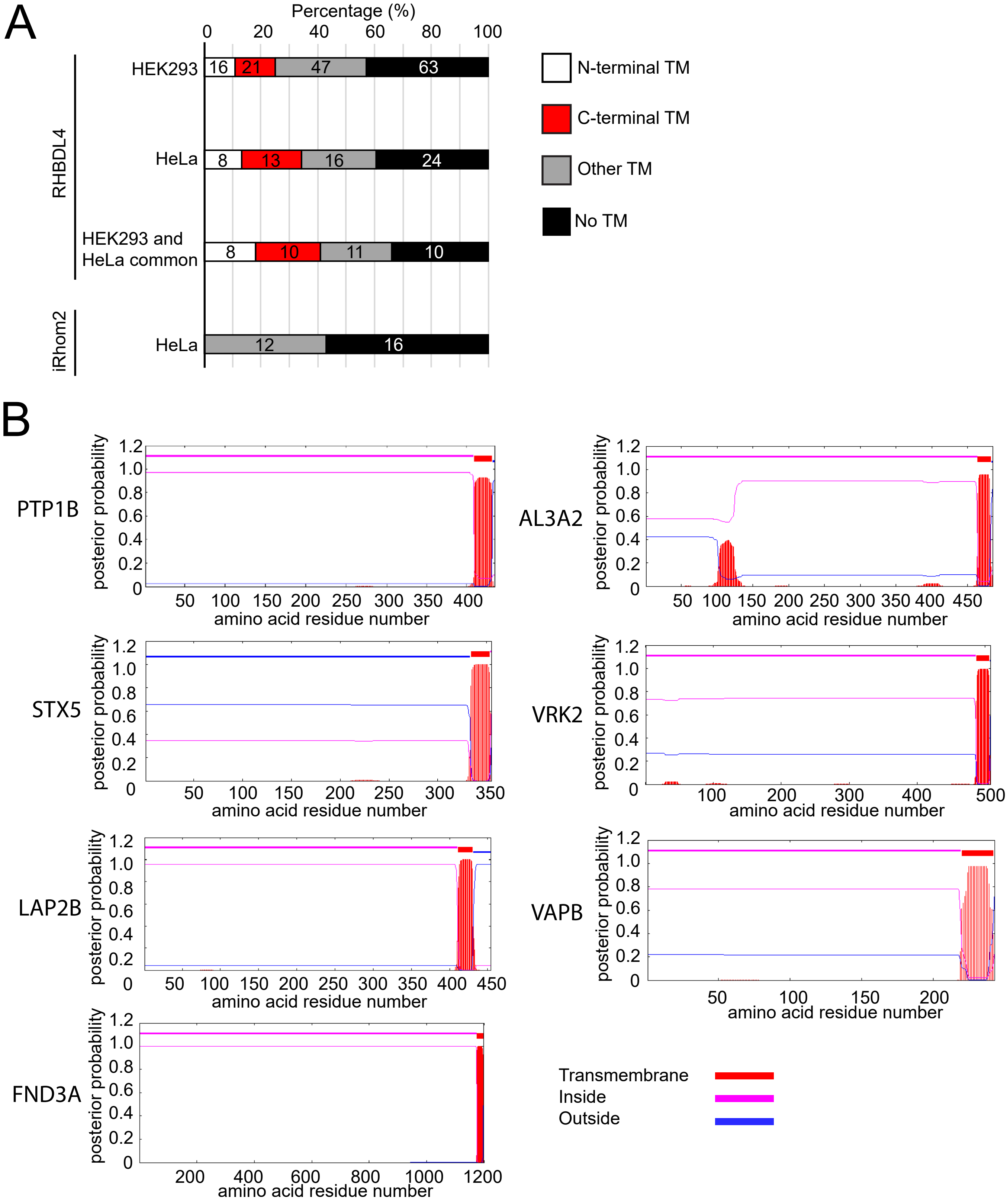

Supporting Information 3 Predicted topology of candidates in the BioID of RHBDL4 and iRhom2 according to TMHMM. A) Percentage of proteins found in BioID of RHBDL4 in HEK293, HeLa, HEK293 and HeLa, or of iRhom2 in HEK293 cells. The graph shows the percentages of proteins with predicted no TM (black), N-terminal TM (white) and TA (red) and other transmembrane (grey).
B) Probability of transmembrane region (posterior probability) along the primary sequence of the predicted TA proteins found in both the BioID of RHBDL4 in HEK293 and HeLa.

```
                                                                                End of TM 6
                                                                                   V
sp|Q8TEB9|RHBL4_HUMAN  NNHYCPGGFVNILGFPVPNRFACWVELVAIHLFSPGTSFAGHLAGILVGLMYTQG----- 208
sp|Q8BHC7|RHBL4_MOUSE  SNHYCPGGFVNILGFPVPNRFACWAELVAIHFCTPGTSFAGHLAGILVGLMYTQG----- 208
tr|E1BT32|E1BT32_CHICK NNHYNPGRVSSVLGLQISSKYACWVELVAIHFIAPRTSFAGHLAGILVGLMYTMG----- 208
tr|Q28H82|Q28H82_XENTR NNYYHPGGSSNVFGILIPNKYACWAELVAIHLLSPGTSFVGHLSGILVGLLYTQG----- 208
tr|Q568J3|Q568J3_DANRE NNHYHPGGATNIMGLPIANRYACWVELVLIHIMNPGTSFVGHLSGILVGLLYTTG----- 208
sp|Q8RXW0|RBL14_ARATH  LNSQA-EDYSSVYGILVPTKYAAWAELILVQMFVPNASFLGHLGGILAGIIYLKLKGSYS 224
tr|Q9AQU7|Q9AQU7_ORYSJ LNAWS-DDYVFLHGVVIPAKYAAWAELLLIQAFIPGTSLIGHLGGILAGLAYLWLKRSFS 238

sp|Q8TEB9|RHBL4_HUMAN  ---PLKKI-------MEACAGGFSSSVGYPGRQYYFNSSGSSGYQDYYPHGRPDHYEE-  256
sp|Q8BHC7|RHBL4_MOUSE  ---PLKKI-------MDTCAGIFISHAGPSGQQNHFNNAGPSGYQNHYADGRPVTYDA-  256
tr|E1BT32|E1BT32_CHICK ---PLKKI-------MKACAGGFSSFADPDRPR--NDYSGYSEYYRY-----PDDQYR-  249
tr|Q28H82|Q28H82_XENTR ---PLKKI-------LISTAS-FSNGPSSTQRN--FSYSGYSGYADD-----SYQQHS--  248
tr|Q568J3|Q568J3_DANRE ---PLKRL-------MEICAGFVTSNGHYGGQQTYYNSSGYSGYGMPYAPNTNYEQHYYG 258
sp|Q8RXW0|RBL14_ARATH  GSDPVTMAVRGVSRLVTWPLRFLNGMVRSRRRR--ITGRGRVGRGQTGIAGPGI------ 276
tr|Q9AQU7|Q9AQU7_ORYSJ GPDPLSLLISGIGKAVRWPVGFVQKLFRSGRPQGYTPSRGRVGRGSARENGRGI------ 292

sp|Q8TEB9|RHBL4_HUMAN  ---------------APRNYDTYTAGLSEEEQLERALQASLWDRGNT----RNSPPPYGF 297
sp|Q8BHC7|RHBL4_MOUSE  ---------------TYRNYDVYTAGLSEEEQLERALRASIWDRGNT----RNGPMPYGF 297
tr|E1BT32|E1BT32_CHICK ---------------TPNNYYEYTGGLTEEEQLERAVLNSLNERDFGGATYNNERRPYGF 294
tr|Q28H82|Q28H82_XENTR ---------------SSRSYDLYTGGFDEDEQLQQAIRESLHERDGHRR-----PQQNAP 288
tr|Q568J3|Q568J3_DANRE APYRQQYNTNSAHTPTAPPQHPYTAGMSEEQQYEAALRASLNDRGGHTQAR----SSHGP 314
sp|Q8RXW0|RBL14_ARATH  --WRC----------QSCTYDN-SGWLSACE-----MCGSGRARGNG-WSLN----QGPA 313
tr|Q9AQU7|Q9AQU7_ORYSJ --WRC----------SACTYDN-SPSTDICE-----MCSSAREDH-A-FSHR----QHLQ 328

sp|Q8TEB9|RHBL4_HUMAN  HLSP-----EEMRRQRLHRFDSQ 315
sp|Q8BHC7|RHBL4_MOUSE  RLPP-----EEMRRQRLHRFDGQ 315
tr|E1BT32|E1BT32_CHICK WFPPEQHSEEEMRRQRLRRFWRQ 317
tr|Q28H82|Q28H82_XENTR WSSAEQLSVEEIRRRRLNRF--- 308
tr|Q568J3|Q568J3_DANRE -FVPSNPSPEEIRWRRIQRFDS- 335
sp|Q8RXW0|RBL14_ARATH  LSSSNDLPLDELRRRRVERFS-  334
tr|Q9AQU7|Q9AQU7_ORYSJ AGGNGEPSVEEIRRRRLERFSR- 350
```

Supporting Information 4 Alignment of the C-terminus of RHBDL4 homologues from *Homo sapiens, Mus musculus, Gallus gallus, Xenopus tropicalis, Danio rerio, Arabidopsis thaliana*, and *Oryza sativa*. End of the 6th transmembrane domain and beginning of the C-terminus domain is indicated by ”V”. **Bold**ed are tyrosine residues.

A

Protein coverage for sp|Q8TEB9|RHBL4_HUMAN

Identified in SUB8791 - HEK293 BioID R4 iR2 / MSS11056 - HEK293 BioID R4 iR2 (Meta)

sp|Q8TEB9|RHBL4_HUMAN - Rhomboid-related protein 4 OS=Homo sapiens GN=RHBDD1 PE=1 SV=1

Percent Coverage: 52.1%

```
MQRRSRGINT GLILLLSQIF HVGINNIPPV TLATLALNIW FFLNPQKPLY SSCLSVEKCY QQKDWQRLLL SPLHHADDWH  80
LYFNMASMLW KGINLERRLG SRWFAYVITA FSVLTGVVYL LLQFAVAEFM DEPDFKRSCA VGFSGVLFAL KVLNNHYCPG 160
GFVNILGFPV PNRFACWVEL VAIHLFSPGT SFAGHLAGIL VGLMYTQGPL KKIMEACAGG FSSSVGYPGR QYYFNSSGSS 240
GYQDYYPHGR PDHYEEAPRN YDTYTAGLSE EEQLERALQA SLWDRGNTRN SPPPYGFHLS PEEMRRQRLH RFDSQ
```

Click here to view PTM summary report for this protein ID.

B

Y264

NYDTY(+79.97)TAGLSEEEQLER

1 x Phospho (Y)

Identified in MSS11093 with qvalue 0.00

Charge: 2, Exp. m/z: 1049.940, Calc. m/z: 1049.437

Data File: Qex03_SVH_150624_kyo_K2, Scans: 32745 - 32745

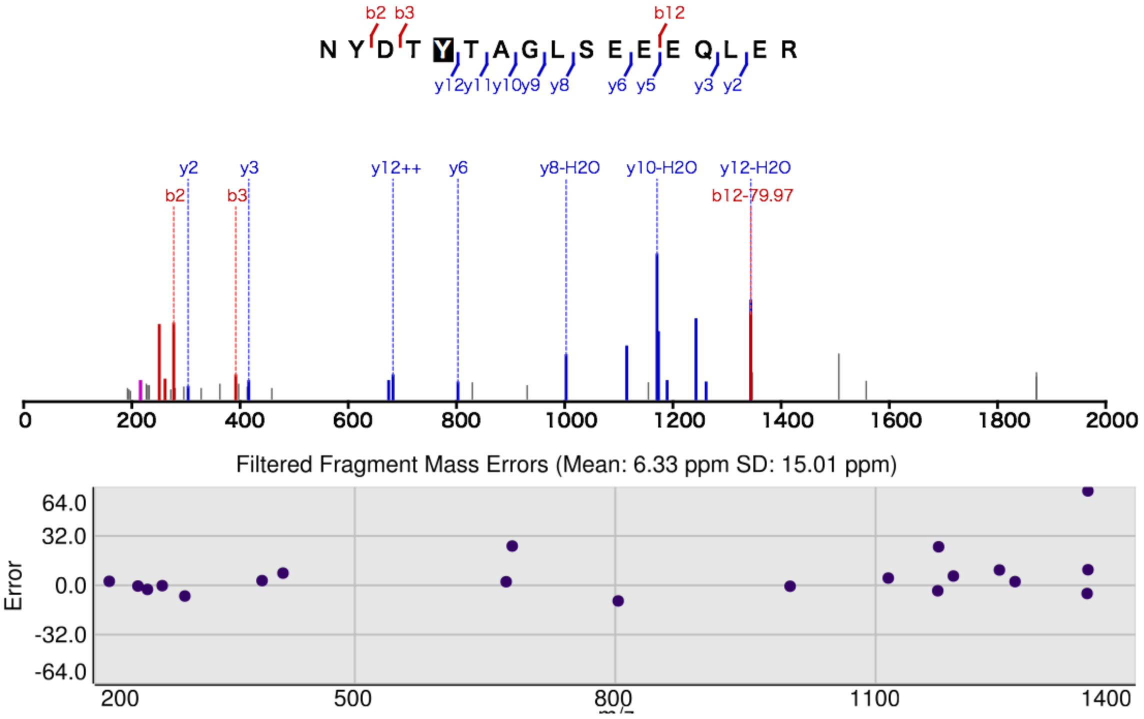

S269

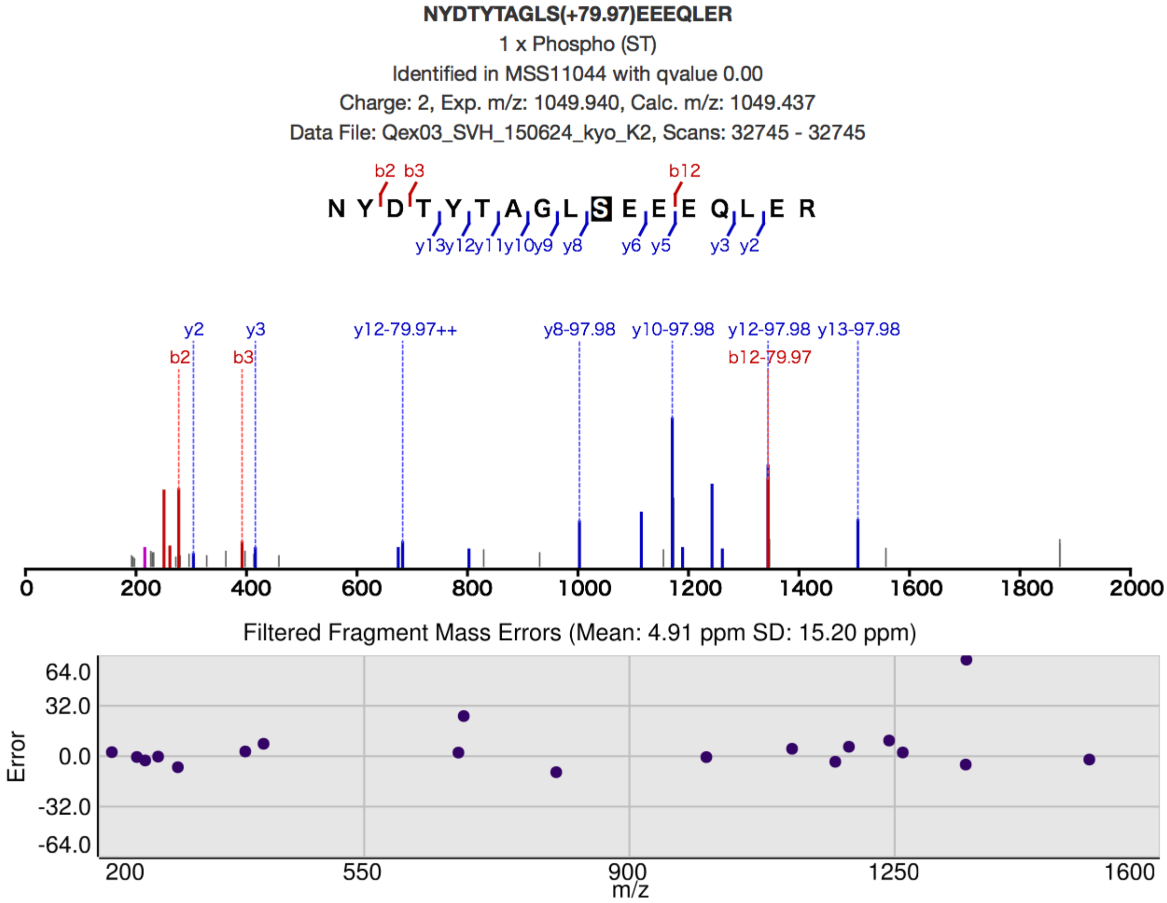
NYDTYTAGLS(+79.97)EEEQLER
1 x Phospho (ST)
Identified in MSS11044 with qvalue 0.00
Charge: 2, Exp. m/z: 1049.940, Calc. m/z: 1049.437
Data File: Qex03_SVH_150624_kyo_K2, Scans: 32745 - 32745
b2 b3 b12
N Y D T Y T A G L S E E E Q L E R
y13 y12 y11 y10 y9 y8 y6 y5 y3 y2
y2 y3 y12-79.97++ y8-97.98 y10-97.98 y12-97.98 y13-97.98
b2 b3 b12-79.97
0 200 400 600 800 1000 1200 1400 1600 1800 2000
Filtered Fragment Mass Errors (Mean: 4.91 ppm SD: 15.20 ppm)
Error
64.0 32.0 0.0 -32.0 -64.0
200 550 900 1250 1600
m/z

T289

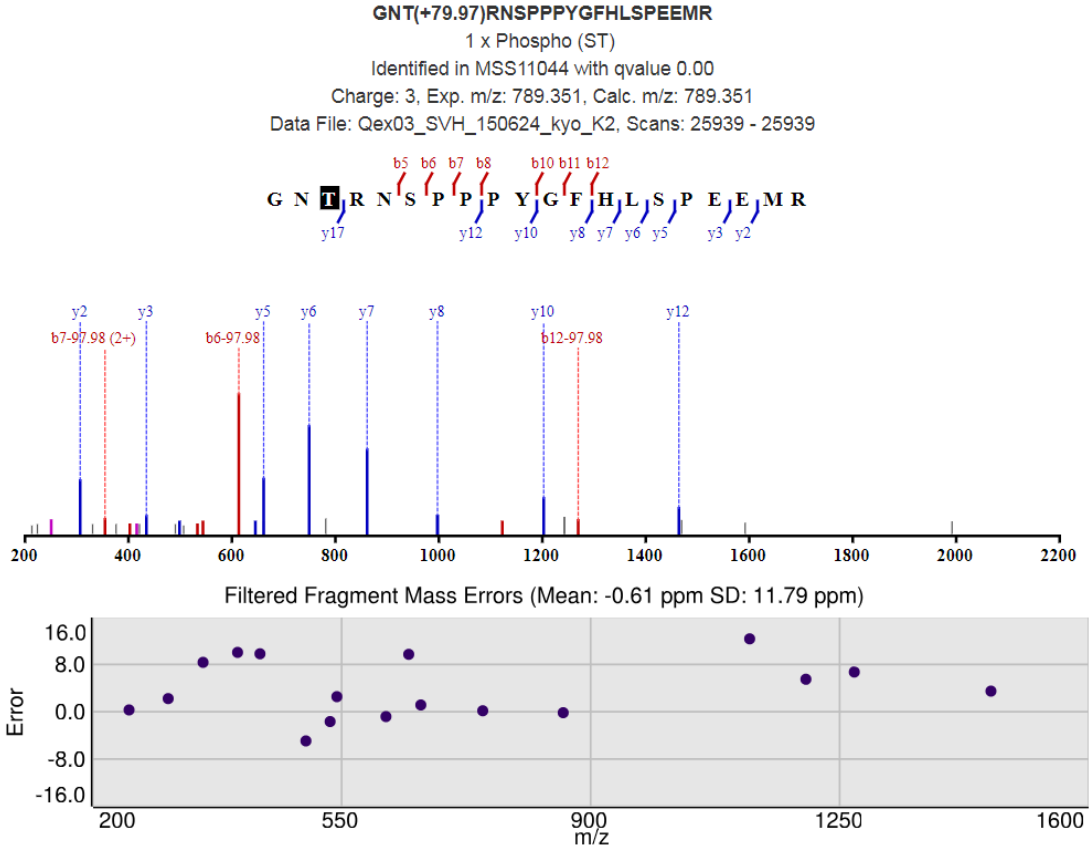
GNT(+79.97)RNSPPPYGFHLSPEEMR
1 x Phospho (ST)
Identified in MSS11044 with qvalue 0.00
Charge: 3, Exp. m/z: 789.351, Calc. m/z: 789.351
Data File: Qex03_SVH_150624_kyo_K2, Scans: 25939 - 25939
b5 b6 b7 b8 b10 b11 b12
G N T R N S P P P Y G F H L S P E E M R
y17 y12 y10 y8 y7 y6 y5 y3 y2
y2 y3 y5 y6 y7 y8 y10 y12
b7-97.98 (2+) b6-97.98 b12-97.98
200 400 600 800 1000 1200 1400 1600 1800 2000 2200
Filtered Fragment Mass Errors (Mean: -0.61 ppm SD: 11.79 ppm)
Error
16.0 8.0 0.0 -8.0 -16.0
200 550 900 1250 1600
m/z

S291

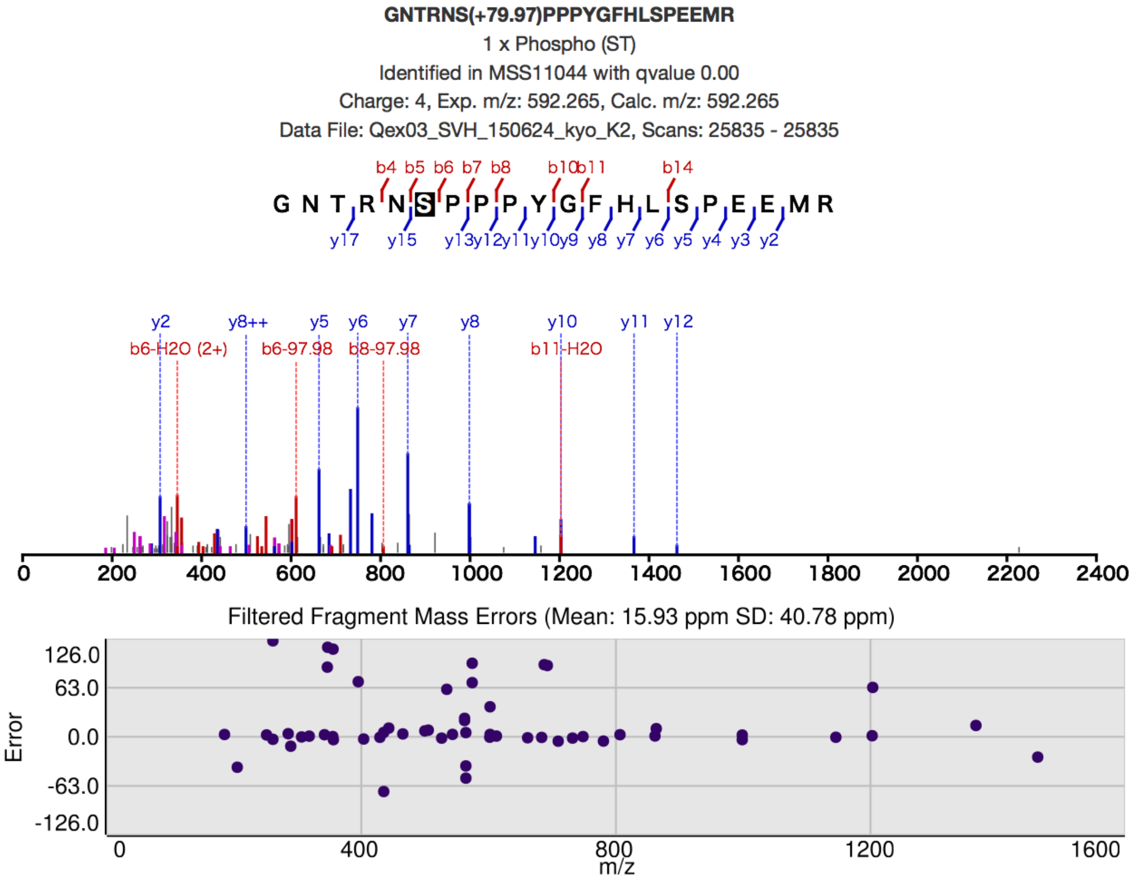
GNTRNS(+79.97)PPPYGFHLSPEEMR
1 x Phospho (ST)
Identified in MSS11044 with qvalue 0.00
Charge: 4, Exp. m/z: 592.265, Calc. m/z: 592.265
Data File: Qex03_SVH_150624_kyo_K2, Scans: 25835 - 25835
Filtered Fragment Mass Errors (Mean: 15.93 ppm SD: 40.78 ppm)
Error
m/z

Y295

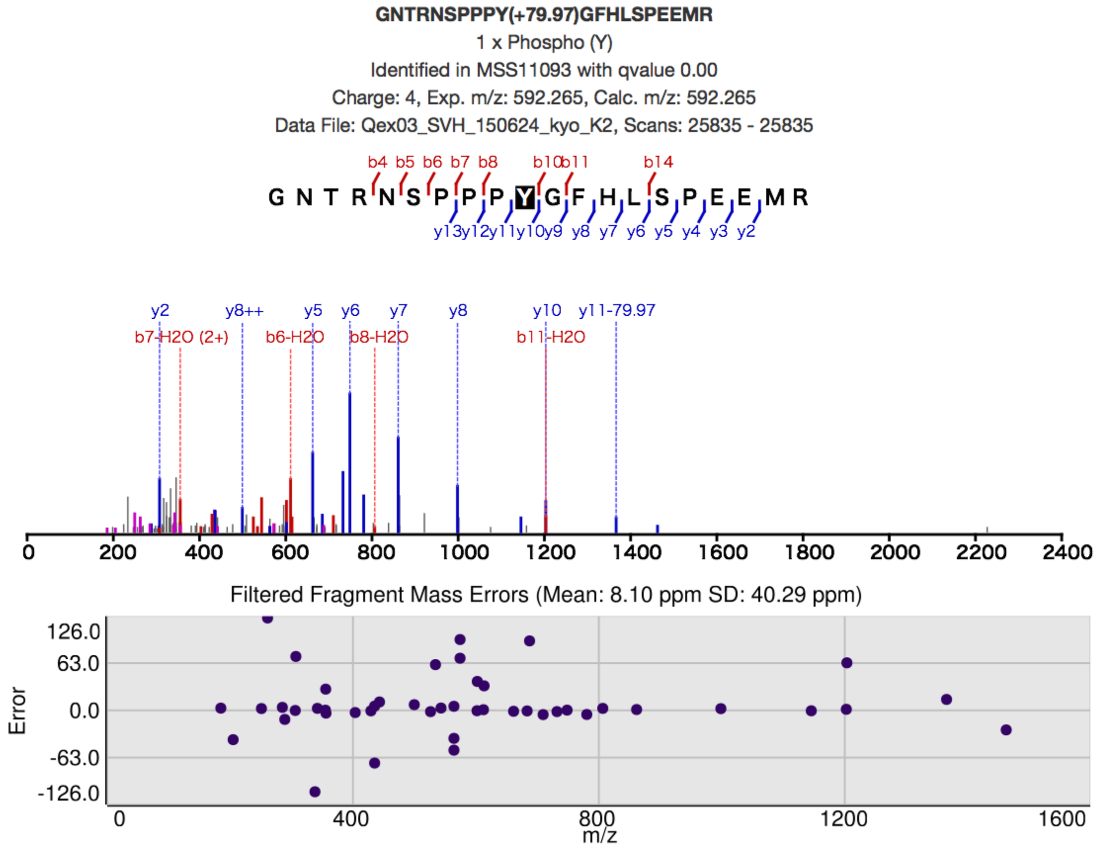
GNTRNSPPPY(+79.97)GFHLSPEEMR
1 x Phospho (Y)
Identified in MSS11093 with qvalue 0.00
Charge: 4, Exp. m/z: 592.265, Calc. m/z: 592.265
Data File: Qex03_SVH_150624_kyo_K2, Scans: 25835 - 25835
Filtered Fragment Mass Errors (Mean: 8.10 ppm SD: 40.29 ppm)
Error
m/z

Y295

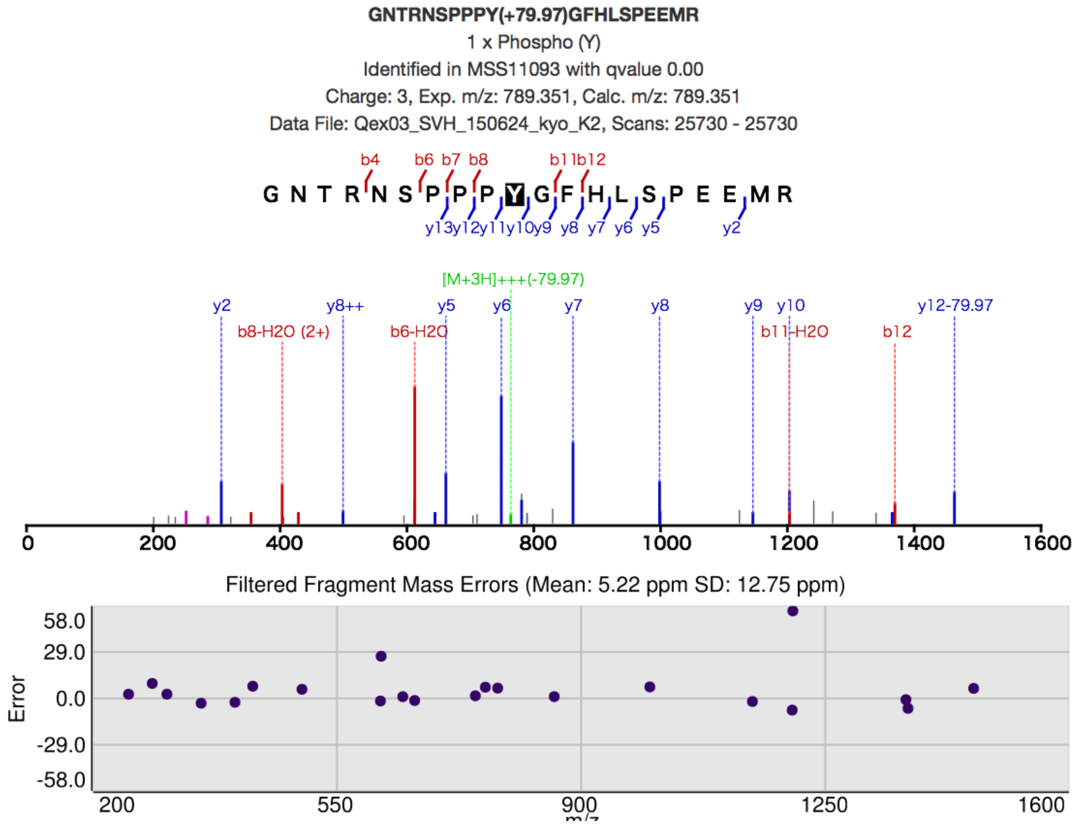

S300

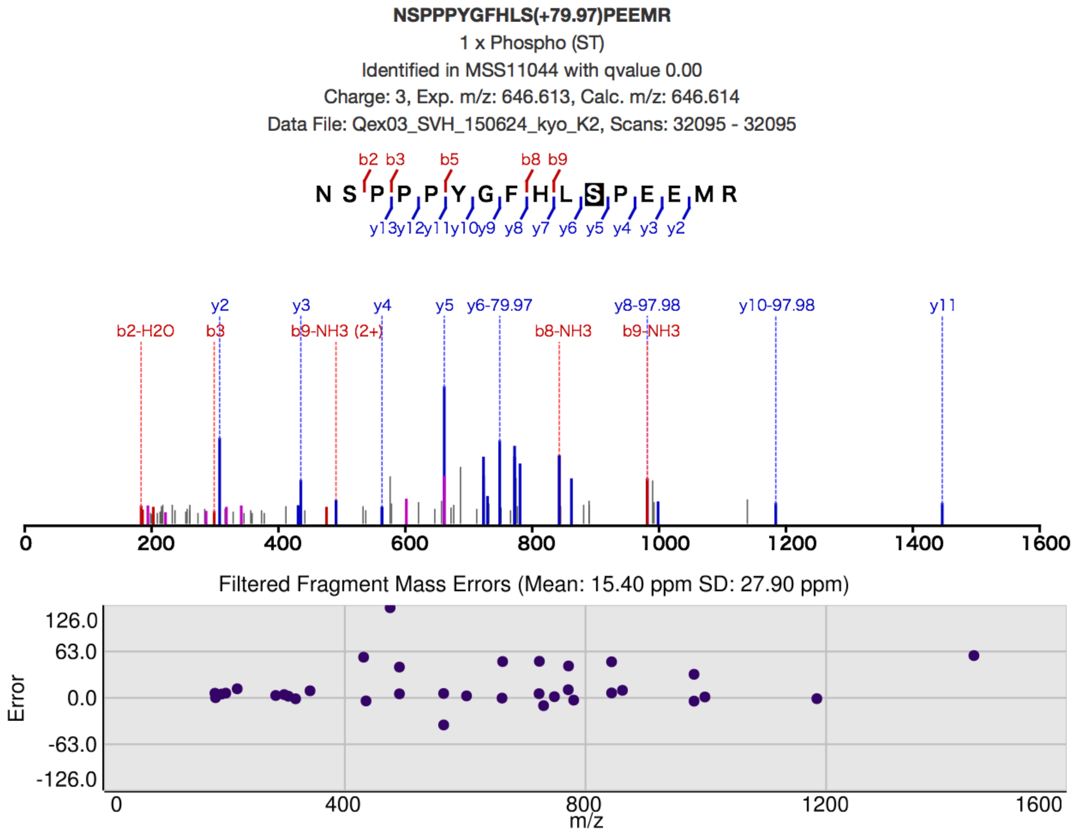

Supporting Information 5 MS coverage and fragmentation of the peptides mapping to RHBDL4. A) Peptides cover 52 % of RHBDL4. B) Fragmentation gallery of phospho-peptides mapping to RHBDL4. All identification are q value <0.01.
